# Investigating the Mechanical, Thermal, and Crystalline Properties of Raw and Potassium Hydroxide Treated Butea Parviflora Fibers for Green Polymer Composites

**DOI:** 10.3390/polym15173522

**Published:** 2023-08-24

**Authors:** Abisha Mohan, Retnam Krishna Priya, Krishna Prakash Arunachalam, Siva Avudaiappan, Nelson Maureira-Carsalade, Angel Roco-Videla

**Affiliations:** 1 PG & Research Department of Physics, Holy Cross College, Nagercoil, Affiliated to Manonmaniam Sundaranar University, Tirunelveli 627012, India; mabisha@proton.me; 2 Department of Civil Engineering, University College of Engineering Nagercoil, Anna University, Nagercoil 629004, India; 3 Departamento de Ingeniería Civil, Universidad de Concepción, Concepción 4070386, Chile; savudaiappan@udec.cl; 4 Centro Nacional de Excelencia para la Industria de la Madera (CENAMAD), Pontificia Universidad Católica de Chile, Av. Vicuña Mackenna 4860, Santiago 8330024, Chile; 5 Department of Physiology, Saveetha Dental College and Hospitals, SIMATS, Chennai 600077, India; 6 Departamento de Ingeniería Civil, Universidad Católica de la Santísima Concepción, Concepción 4090541, Chile; 7 Facultad de Salud y Ciencias Sociales, Universidad de las Américas, Providencia, Santiago 7500975, Chile

**Keywords:** green composites, stem fiber, crystallinity, thermal behavior, reinforcement material

## Abstract

The only biotic factor that can satisfy the needs of human species are plants. In order to minimize plastic usage and spread an immediate require of environmental awareness, the globe urges for the development of green composite materials. Natural fibers show good renewability and sustainability and are hence utilized as reinforcements in polymer matrix composites. The present work concerns on the usage of Butea parviflora fiber (BP), a green material, for high end applications. The study throws light upon the characterization of raw and potassium hydroxide (KOH)–treated Butea Parviflora plant, where its physical, structural, morphological, mechanical, and thermal properties are analyzed using the powder XRD, FTIR spectroscopy, FESEM micrographs, tensile testing, Tg-DTA, Thermal conductivity, Chemical composition, and CHNS analysis. The density values of untreated and KOH-treated fibers are 1.238 g/cc and 1.340 g/cc, respectively. The crystallinity index of the treated fiber has significantly increased from 83.63% to 86.03%. The cellulose content of the treated fiber also experienced a substantial increase from 58.50% to 60.72%. Treated fibers exhibited a reduction in both hemicelluloses and wax content. Spectroscopic studies registered varying vibrations of functional groups residing on the fibers. SEM images distinguished specific changes on the raw and treated fiber surfaces. The Availability of elements Carbon, Nitrogen, and Hydrogen were analyzed using the CHNS studies. The tensile strength and modulus of treated fibers has risen to 192.97 MPa and 3.46 Gpa, respectively. Thermal conductivity (K) using Lee’s disc showed a decrement in the K values of alkalized BP. The activation energy E_a_ lies between 55.95 and 73.15 kJ/mol. The fibers can withstand a good temperature of up to 240 °C, presenting that it can be tuned in for making sustainable composites.

## 1. Introduction

For centuries, the distinctive characteristics of natural fibers have made them valuable for diverse purposes. The properties of natural fibers, including their mechanical, physical, and chemical attributes, are contingent on factors such as the specific fiber type, the plant species from which they are derived, and the environmental conditions in which they are cultivated. Natural fibers are categorized based on their chemical composition, which can be either cellulose-based or lignin-based. Cellulose-based fibers such as cotton, jute, flax, hemp, and sisal have high tensile strength, suitable flexibility, and low density, making them suitable for applications such as textiles, paper, and composites. Lignin-based fibers, such as wood fibers, have high stiffness and strength, making them suitable for applications such as building materials and composites. Due to their environmentally friendly and sustainable behavior, natural fibers are progressively being utilized as substitutes for synthetic fibers in a wide range of applications. The lowered density of natural fiber composites (NFCs), along with their advantageous tribological and insulating qualities, could increase the cargo capacity of aircraft. Boeing and Airbus, two aviation industry titans, applied considerable effort to learn more about the usage of natural fibers in airplane interiors [1].

Natural fiber composites (NFCs) are composite materials that are made from a combination of natural fibers and a matrix material. NFCs are becoming increasingly popular as a sustainable and environmentally friendly substitute for conventional composite materials, which predominantly rely on synthetic fibers. The natural fibers used in NFCs can come from plant, animal, or mineral sources. The matrix material can be made from a variety of materials such as bio-based polymers, thermosetting resins, or thermoplastics. The characterization of natural fibers are instrumental in developing and optimizing new applications. NFCs have numerous advantages over traditional composite materials. The efficient properties possessed by natural fibers are light weight, high aspect ratio, low density, soundproof, thermal, mechanical properties, and biodegradability [2,3,4]. The combined effect of cellulose, hemicellulose, lignin, and wax dictates the overall properties of fibers. However, the hydrophilicity of the fibers turn in as a threat while intriguing fibers in making composites [5,6]. Microwave drying systems using halogen lamps were employed to bring down the moisture absorption in bast fibers [7]. The inadequate interfacial bonding contributes to diminished mechanical properties, which can be influenced by factors such as contact angle, orientation of microfibrils relative to the cell axis, and the Young’s modulus of the fiber [8]. By subjecting fibers to different treatments, it is possible to transit their hydrophilic nature to hydrophobic, resulting in improved performance and easier disposal [9,10,11]. Studies in the literature demonstrate that alkali treatment has caused notable changes in the mechanical properties of reinforcements [12,13,14,15]. Specifically, alkali–treated Borassus fruit fibers exhibited significant increase of 41% in tensile strength, 69% in modulus and 40% in elongation [16]. 5% NaOH action on Acacia Caesia bark fibers had removed amorphous constituents and improved its tensile nature [17]. KOH–treated Ijuk fibers displayed enhanced tensile and stiffness in the fabricated composites [18]. Furthermore, natural fibers typically require lower processing temperatures compared to synthetic alternatives, which could be overthrown by employing flame retardants like phosphates, phosphoric acids, N-methynol functional phosphorus esters, antimony-halogen combinations, boron and nitrogen compounds [1]. Generally practiced chemical treatment process are bleaching, benzoylation, acetylation, silane, permanganate, etc. 

The present work focuses on the Butea Parviflora (BP) plant, which is native to most South East Asian countries, including India. It is one among the many plants of the Fabaceae family with the genus name ‘Butea’. It has a trifoliate alternate spiral leaf arrangement and bears flowers and seeds. Seeds are imbibed with many pharmaceutical benefits and are crushed for oil [19]. Being a deciduous climbing shrub, it could extend up to 20 m in height. Long fiber strands are torned out for domestic utilities by localities.

The Butea parviflora (BP) fiber is believed to possess most characters, as found in other stem fibers discovered to date, and there has been limited research conducted on it. The BP plant has climbing branches twined strongly around each other. The roots are strongly fixed to the ground, thus rendering a mechanical support from retrieving its path of growth. They are scattered all over India mostly in the Western and Eastern ghats and are widely flourished from moist to arid region. Fibers for the present study are collected from the village of Thirunandikarai, Kanniyakumari District, Tamil Nadu. Characterization of BP fibers is necessary to understand their properties and potential applications. Raw and 0.1 M of KOH–treated Butea fibers are prepared for the characterization procedures including physical, mechanical, spectroscopic, thermal, crystalline, morphological, and chemical testing methods. Physical testing methods involve measurements of fiber diameter, length, density and aspect ratios. Mechanical testing techniques are employed to assess the strength and rigidity of fibers, while the spectroscopic, thermal, crystalline, and morphologies are studied using the FTIR, Thermogravimetric analysis (TG-DTA), X-ray diffraction (XRD), and Scanning Electron Microscopic studies (SEM) [20]. Chemical testing methods are used to identify the chemical composition of fibers, including the detection of impurities and extractives. The experimental data indicate that BP has the potential to serve as a superior reinforcing material in the formulation of sustainable composites.

## 2. Materials and Methods

### 2.1. Material Extraction

**Figure 1:**
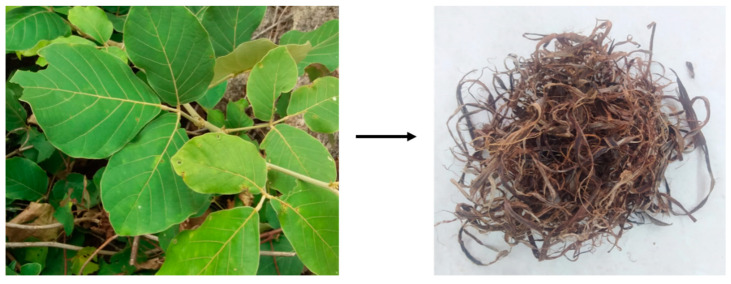
Collected fibers from Butea parviflora plant.

The collected BP fibers are mechanically removed from the branchy stems using a metal teeth. The peeled fibers are then dried in the absence of sunlight for about 7 days in a clean environment. Fibers are drenched in water and surface modification is attained by soaking it in 0.1 M of KOH environment for 30 min. Alkali pre-treatment is performed to eliminate impurities such as wax, oil, etc., from the fibers, while also inducing modifications to enhance their properties [21,22]. Fibers are kept at room temperature for over 10–15 days. Potassium hydroxide was chosen over NaOH in the current study, because it is less alkaline. In Ijuk fibers, KOH–treated fibers generated the highest tensile and stiffness than NaOH [18]. Given that KOH treatment was not performed prior on Butea fibers, KOH with 0.1 molarity was carried on Butea fibers. Fibers mercerized with 0.1 M KOH solution on other fibers demonstrated an enhancement in the mechanical properties [23]. Sisal fibers from the literature showed an improved hydrophobic behavior while treated with same molarity of alkali solution [24]. Alkali-treated fibers are proceeded with vacuum desiccating for 2 days [25]. Figure 1 shows the fibers extracted from Butea parviflora (BP).

### 2.2. Physical Properties of Butea Parviflora (BP) Fiber

The physical factors of unprocessed and alkali–treated Butea parviflora (BP) fibers are crucial in making composites. Randomly selected BP fibers 30 in number are taken to establish the physical aspects.

Diameter of BP is calculated using an optical microscope. KOH–treated fibers show a decrease in the diameter of the raw fibers. It can be believed that the interfacial strength decreases with an increase in diameter regardless of surface modifications [26].

Aspect ratios of natural fibers are found by calculating the ratio between the length and diameter. The aspect ratios of alkali–treated BP (203.91) are greater than raw BP (174.59). Higher the aspect ratios, more will be the compressive strength of composites. The addition of coconut and oil palm fibers to soil building blocks resulted in an augmentation of both compressive and tensile strength, which is correlated with higher aspect ratios of the fibers [27]. 

The linear density (LD) is a measure to determine the fineness of a fiber, and excellency in tensile strength is observed with higher LD values. An average length of 10 cm was chosen to calculate the fiber’s LD using the equation [28].

(1) $$\mathrm{Linear}\;\mathrm{density}\;(\mathrm{LD})=\frac{massoffibers\;(grams)}{lengthoffibers\;(meter)}$$

Density plays a crucial role in determining the suitability of natural fiber composites for various applications. It is a prime factor that distinguishes and discriminates natural fiber composites from their synthetic counterparts. Density is analyzed using the liquid pycnometer method, with the immersion liquid toluene, using the equation [9,29],

(2) $$\rho =\frac{\left(mb-ma\right)}{\left[\left(mc-ma\right)-\left(md-mb\right)\right]}\rho t$$

In the given context, ma represents the mass of the empty pycnometer (in kilograms), mb denotes the mass of the pycnometer filled with fibers (in kilograms), mc represents the mass of the pycnometer filled with toluene (in kilograms), and md indicates the mass of the pycnometer filled with both fibers and toluene (in kilograms).

The density of KOH–treated BP (1.340 g/cc) is higher than raw BP (1.238 g/cc). Less-dense extractives of fibers like lignin and hemicellulose, along with airspaces, might get removed by the alkalization. Hence, the density of treated BP has been incremented [28]. Density values of BP are comparable with other fibers like Thespesia populnea (1.412 g/cc) [30], carbon (1.40 g/cc), and aramid fibers (1.40 g/cc) and are much smaller than E-glass fibers (2050 g/cc) [31]. Physical aspects of Butea fibers are displayed in Table 1.

## 3. Characterization Studies 

### 3.1. X-ray Diffraction (XRD) Analysis

The crystalline nature of Butea fibers was measured using powder X-ray diffraction. The analysis was conducted using a D8 Advance Model diffractometer from the manufacturer, Bruker AXS, Karlsruhe, Germany. Recording the spectrum for 2θ values was taken between 3° and 80° under 40 kV and a current supply of 35 mA. The Segal empirical formula was utilized to calculate the crystallinity index of BP fibers [34,35].

(3) $$\mathrm{CI}=\frac{I_{200-I_{am}}}{I_{200}}*100\%$$

where I_200_—maximum intensity of the crystalline diffraction peak at 2θ angle range of 22° to 23°, and I_am_—minimum intensity of an amorphous peak at 2θ angle of 18°. Additionally, the crystallite size was calculated utilizing Scherrer’s equation [36].

(4) $$\mathrm{CS}=\frac{K\lambda }{\beta_{200}cos\theta }$$

where K—Scherrer’s constant, λ—wavelength of X-rays (0.154 nm), β_200_—the peak’s full width at half maximum, and θ—Bragg angle.

### 3.2. Scanning Electron Microscopy (SEM)

Scanning electron microscopy gives outstanding results in identifying the morphological features; thereby, the fundamental characters of the fibers are lit up with detailed clarity. The surface images of fibers were scanned with the working voltage from 0.5 to 30 kV, using an instrument, Jeol 6390LA/OXFORD XMXN, from JEOL India PVT LTD; South Delhi, India, a subsidiary company of JEOL Limited, Tokyo, Japan. 

### 3.3. Thermogravimetric Analysis

Heat resistance is very much needed for making composites [37]. By indulging fibers in thermal analysis, the nature of samples under various environments of heating and cooling, along with inert oxidation-reduction atmospheres, can be cited. The change in mass is adjoined with a variety of reactions such as decomposition, degradation, adsorption, vaporization, oxidation, reduction, etc. Tg-dta and DSC analyses were carried out using the Perkin Elmer STA 6000 Model, from the manufacturer Perkin Elmer Inc., Mumbai, India. The heating process was monitored at a rate of 20 °C per minute under a dynamic nitrogen atmosphere within the temperature range of 40–800 °C.

### 3.4. Thermal Conductivity Using Lee’s Disc Method

Thermal conductivity was assessed using Lee’s disc method, wherein the mass, diameter, and thickness of Lee’s disc were measured using a digital weighing machine, Vernier caliper, and screw gauge. At the onset of steady temperature, the disc is let to cool down, and dropping temperatures are noted. The thermal conductivity was determined by employing a specific equation for the calculation process [38].

(5) $$k=\frac{mxd\left(r+2h\right)}{{\pi r}^{2}\left(T1-T2\right)\left(2r+2h\right)}dT/dt\;W/m/K$$

The various parameters involved are: m represents the mass of the Lee’s disc, d refers to the sample thickness, x denotes the specific heat, r represents the radius of the Lee’s disc, h signifies the thickness of the Lee’s disc, and *dT/dt* represents the tangential slope. Additionally, T1 represents the steady temperature of the vapor chamber, and T2 represents the steady temperature of the Lee’s disc.

### 3.5. CHNS Analyzer

CHNS elemental analysis offers a quick method to determine the levels of carbon, hydrogen, nitrogen, and sulfur in organic samples and various other materials, including volatile or viscous samples. The analysis was performed using the model Elementar Vario EL III, Micro Cube manufactured by Elementar, Langenselbold, Germany with a precision > 0.1% absorbance.

### 3.6. Single Fiber Tensile Testing

The tensile strength of BP fibers were measured using single fiber strength and elongation (Zwick/Roell) from the Physical Testing Laboratory, SITRA, Coimbatore. All analyses were conducted at a controlled temperature of approximately 21 °C with a tolerance of ±1 °C, along with a relative humidity of 65%. The gauge length was set at 50 mm, and the transverse rate was maintained at 30 mm/min. The tensile strength of BP fibers was determined using [39]

(6) $$Tensile\;strength\left(\sigma \right)=\frac{Tensile\;force\;(F)}{cross\;sectional\;area\;of\;fibers\;(A)}$$

The microfibril angles of BP fibers are calculated using the global deformation equation [40].

(7) $$ԑ=\ln\;(1+\frac{\Delta L}{L})=-ln\;(\cos\alpha )$$

where ε—strain developed, α—microfibril angle (MFA), L—fiber length, and ΔL—elongation at the time of breaking.

### 3.7. FTIR Analysis

The FTIR spectrometer (Model FTIR-8400S spectrum, SHIMADZU, Kyoto, Japan) was employed to identify the functional groups present in both untreated and alkali–treated fibers. The analysis was conducted using a KBr matrix with a scan rate of 45 scans per minute and a resolution of 4 cm^−1^, within a wavenumber range of 400 cm^−1^ to 4000 cm^−1^.

## 4. Results and Discussion

### 4.1. Determination of Chemical Composition 

The presence of cellulose, lignin, hemicellulose, and wax content in the fiber sample was determined through chemical analysis. Extraction methods, maturity of plant parts, and the habitat of plants would have a direct outcome on the cellular compositions [41]. Standardized methods were followed to find the cellular composition. Percentage of cellulose and hemicellulose was found from the acid and neutral detergent method. Lignin content was found using the Klason method, and moisture quantity was measured by drying the sample. The wax percentage was determined using the Soxhlet extraction method, where the chosen solvent’s vapor dissolves wax from the fiber samples. The variance between the extracted mass and the dried mass calculates the wax% present in the samples.

The cellulose content of 0.1 M KOH–treated BP was 60.72%, which is higher than the raw fiber (58.5%) and is thought to withstand hydrostatic pressure gradients of the fibers. After alkali treatment, fibers showed a visible improvement to serve as reinforcement material [42]. The cellulose values are in agreement with Kenaf (53.14%) [43] and Okra fibers (60–70%) [44]. Hemicellulose in alkalized BP deeply declined to 19.2% from 40.13%. There are almost no comprehensive treatment methods to extract hemicellulose completely without dissolving the cell components [45]. Complete removal of hemicelluloses could potentially lead to a reduction in composite strength while enhancing its stiffness [46]. Molecular weights of hemicellulose are lower than cellulose, and also, the alkali treatment on BP has eliminated a high degree of hemicelluloses, and because of that, physical properties such as density, aspect ratio, and linear density show an increase [47].

Furthermore, the complete removal of hemicellulose or lignin through alkalization may not be foolproof due to the presence of hydrogen bonding between residual hemicellulose and cellulose fibrils [48,49]. Lignin contributes to the structural integrity of fibers. A higher lignin percentage (18.09%) of BP can possibly favor excellent rigidity compared to other fibers. The physical properties of BP fibers were not negatively influenced by lignin. However, the presence of lignin impacted the thermal stability of BP fibers by stretching its degradation temperature [50]. The cellulose/lignin ratio in BP fibers was almost around 3:1. It is necessary to obtain a high cellulose/lignin ratio in samples to receive better crystalline, structural, and physical properties while introducing these fibers for composite making [51]. Modifying the cellulose/lignin ratio through diverse oxidative treatments is essential for these fibers, as this approach could yield improved fiber properties, namely (higher thermal stability, high mechanical strength), beyond those observed in the current study. 

The amount of wax housed in the BP fibers (0.31%) was minimized to 0.25% using KOH action, and hence, initial flushing of samples prior to alkali treatment was considered optional. Dewaxing occurred during the alkali action had introduced a rough surface, which is shown in the SEM images. Moreover, the elimination of wax and other contaminants contributed to the enhancement of the tensile properties of the BP fibers [52]. A comparison of the chemical composition between BP fibers and other natural fibers is presented in Table 2 [40].

### 4.2. X-ray Diffraction (XRD) Analysis 

**Figure 2:**
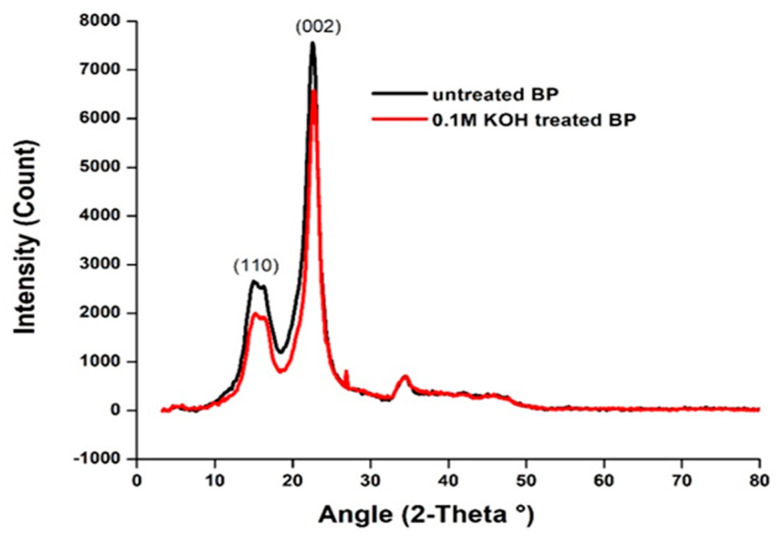
X-ray diffractogram of raw and 0.1 M KOH–treated Butea parviflora (BP).

The XRD analysis revealed the crystalline nature of the BP fibers in Figure 2. The lattice planes at (110) and (200) belong to the crystallographic plane group of celluloses [44]. It turns out that the crystallinity index (CI) of 0.1 M KOH–treated BP (86%) was more than the untreated BP (83%). SEM images also display an ordered arrangement of cellular components in the alkalized fiber. High CI indicates a better orientation of cellulose around the fiber axis, which attributes to the higher strength of fibers [53]. Additionally, the thermal degradation of fibers is also toggled to higher temperatures with the rise in CI. The CI for BP fiber is greater than other fibers and is tabulated in Table 3. Under certain conditions, it is possible for the crystalline regions to undergo rearrangement, leading to an increased level of crystallinity in the fiber [54]. Meanwhile, the crystallite size of the alkalized BP has risen from 7.5 nm to 8.04 nm. The CS of Butea fibers is smaller than the Sida cordifolia stem (18 nm). The increment of CS in the treated BP is suspected owing to the varying strain caused by the intrusion of K^+^ ions on the cellular arrangement during treatment [39].

### 4.3. CHNS Analysis

The presence of elements like carbon, nitrogen, hydrogen, and sulfur in Butea fibers can be detected using the CHNS analyzer. The analysis employs finely chopped raw and alkali-treated fibers. Samples with a high carbon content are regarded advantageous when used as fillers in strengthening composites [12]. The low heat conductivity values obtained from Lee’s disc setup of alkalized BP can be accredited due to its high carbon content. Table 4 shows the weight percent of carbon, hydrogen, and nitrogen.

### 4.4. FESEM Analysis

**Figure 3:**
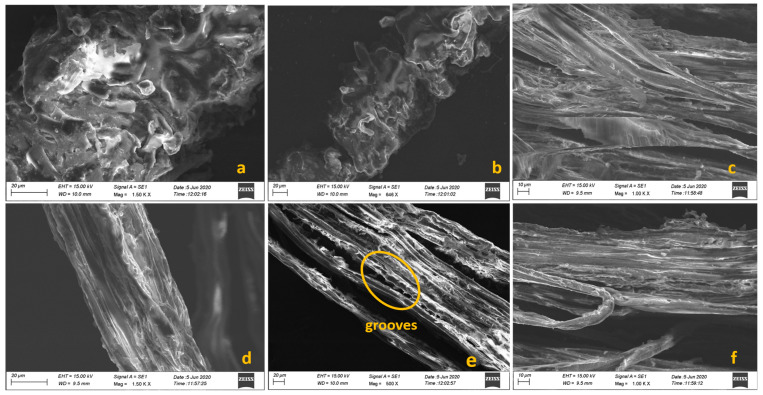
(**a**–**c**) SEM photographs of raw BP, (**d**–**f**) SEM photographs of 0.1 M KOH treated BP.

The surface characteristics of both untreated and 0.1 M alkali-treated BP fibers are depicted in Figure 3a–f. SEM analysis is highly used to question the failure approach at the micro level [57]. The presence of small peaks against the long stripes is seen in the raw fiber. Epidermal projections appear on the longitudinal surface. The clouded irregularities in Figure 3a could be part of non-cellulosic debris [58]. This imperfection is removed in the alkalized fibers. It is assumed that the KOH treatment has washed away most of the oil and waxy impurities tied up with the microfibrils, generating a rough interface on the top of the fibers. The elimination of non-cellulosic structures, mainly wax and hemicellulose, could have created fine grooves along the axis. This might greatly improve the expansive adhesion with the matrix interface [41]. The axial arrangement of treated fibrils is more coordinated than the raw fiber.

### 4.5. Thermogravimetric Analysis

**Figure 4:**
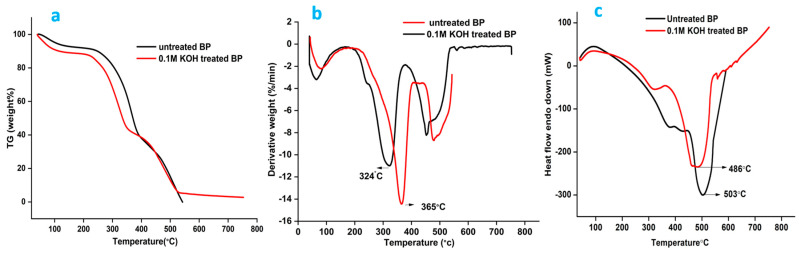
(**a**). Thermogravimetry plot of untreated and alkalized Butea fiber; (**b**). differential thermogravimetry plot of untreated and alkalized BP; (**c**). differential scanning calorimetry curve of raw and alkalized BP.

The thermal nature of BP was monitored between 40 and 800 °C at a heating rate of 20 °C/min. The Tg-dta and DSC curves are provided in Figure 4a,b. Three-step thermal degradation was observed in both fibers. The initial stage of mass loss is anticipated due to the evaporation of moisture present in the fiber [59,60]. The degradation pattern observed in the dtg graph of both fibers between 200 and 260 °C is because of the elimination of hemicellulose. The quick dismissal of cellulose occurs around 240–350 °C leaving anhydro cellulose and levoglucosan [61]. A mass loss of 50 and 45.06% was registered for the raw and treated BP in the second stage, which concerns the exclusion of hemicellulose, lignin, and a tiny fraction of celluloses. The swift reaction is cascaded to the next step with the huge dismissal of hemicellulose. Lignin degradation is registered between the range 280 and 500 °C [62]. Patterns of mass loss noted around specific temperatures are shown in Table 5.

DTG shows that the maximum degradation peak for the alkali-treated fibers has been backtracked to 324 °C compared to that of the raw fiber, which was marked at 365 °C. Alkali action might have dismissed lignin, and hence, the treated fibers have noticed an early decomposition. Minor peaks were noted for the raw and alkalized BP between 400 and 500 °C. Removal of lignin could have occurred within this limit. Weight loss of fibers was stabilized around 500 °C leaving the residues [63]. Other cellulosic fibers like Eucalyptus grandis and Pinus taeda spotted their maximum decomposition temperatures at 353 °C and 360 °C [61].

### 4.6. Differential Scanning Calorimetry

The DSC curves are plotted in Figure 4c. As the temperature increases, notable peaks appear, signaling various thermal events or transitions taking place within the fiber. A prominent endothermic peak was obtained for the KOH-treated fibers at 486 °C. It indicates the pyrolysis and exclusion of lignified compounds, leaving behind char. For the untreated profile, a peak was spotted at 503 °C, owing to the loss of diversified functional groups of lignin. This peak value clearly correlates with the elevated decomposition temperature indicated in the DTG curve. A minor peak was spotted at 360 and 330 °C in the raw and treated BP, marking the removal of cellulose and hemicelluloses. A small hump seen initially around 100 °C in both fibers is because of moisture removal [64]. All the outcomes show that BP fiber can be signed in for making fiber reinforcement composites as long as its thermal stand-by temperature does not exceed 240 °C. 

### 4.7. Activation Energy of Fibers

The kinetic activation energy (Ea) of BP was determined using the Coast–Redfern method [65].

(8) $$\log\left[\frac{-\log\left(1-\alpha \right)}{T^{2}}\right]=\log\frac{\mathrm{AR}}{\;\beta \mathrm{Ea}}\left[1-\frac{2\mathrm{RT}}{\mathrm{Ea}}\right]=\frac{\mathrm{Ea}}{2.303\mathrm{RT}}$$

**Figure 5:**
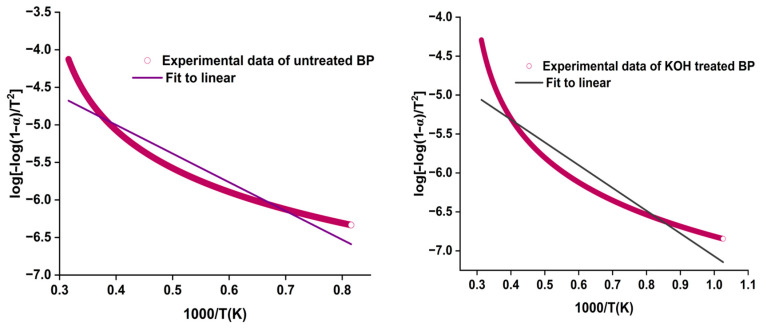
Ea curve of raw and KOH-treated BP.

Ea was estimated through linear interpolation of data points between log[−log(1 − α)/T^2^] and 1000/T. The plot is shown in Figure 5. It speaks more about the aptness of the fibers to be used in composite making. Ea of cellulose fibers show different patterns due to variations in the fiber contents and structure [66].

The activation energy calculated for the raw BP (Ea = 73.15 kJ/mol) was higher than for alkalized fiber (Ea = 55.95 kJ/mol). The activation energy has its impact more on the untreated fiber rather than the alkalized BP. The thermal stability of green fibers is primarily determined by their decomposition temperature. The kinetic activation energy (Ea) values of other fibers are: Prosopis juliflora (76.72 kJ/mol), C. quadrangularis (74.18 kJ/mol), and Coccinia grandis (82.3 kJ/mol) [33,67]. The thermal outcomes of Butea fibers are shown in Table 6.

### 4.8. Thermal Conductivity

**Figure 6:**
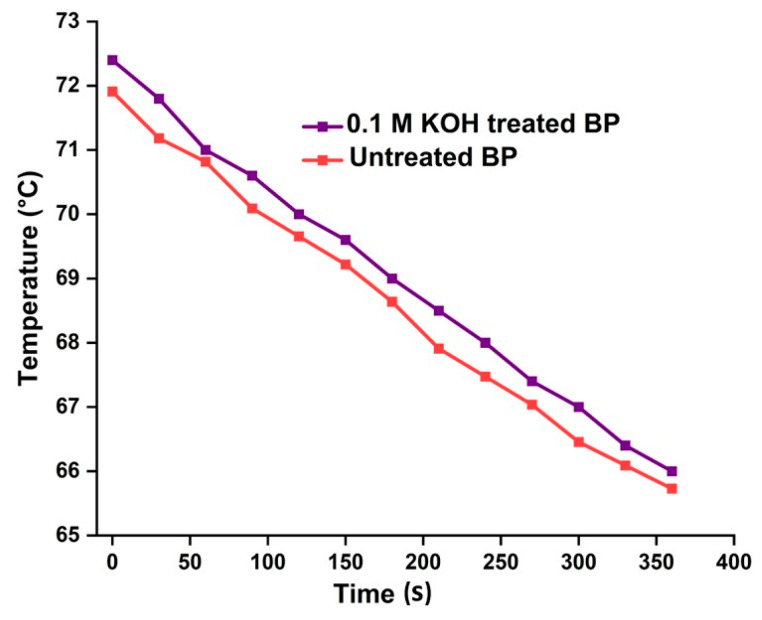
A linear plot of heat transport of raw and KOH-treated BP fibers.

Natural fiber-based materials are highly influential because of their potential insulation behavior. The thermal conductivity (K) of untreated and alkalized BP fiber, found using Lee’s disc method, was K = 0.029 Wm^−1^k^−1^ and K = 0.020 Wm^−1^k^−1^. Thermal conductivity plots of BP fibers are shown in Figure 6. K values of BP fibers are much lower than wood-based thermal insulation foam (k = 0.038 Wm^−1^k^−1^) [68]. The activity was performed at two Lee’s disc setups at room temperature, with the fibers woven tightly without void spaces. The steady temperature of the untreated and 0.1 M KOH-treated fibers are at 73.5 °C and 67.4 °C. Based on the observations, it can be deduced that as the material thickness decreases, its conductivity also reduces, resulting in improved thermal insulation properties [69]. 

The K value of the treated BP fibers is comparatively lower than other plant fibers, like corn stalks (K = 0.121 Wm^−1^k^−1^) and Areca husk fiber (K = 0.021 Wm^−1^k^−1^) [70]. The reduced K value of the alkalized BP accounts for the amorphous content dwelling in the fiber. Lowered heat conducting behavior of BP fibers may lay a path to act as a better thermal insulator, or it can appease the synthetic thermal insulators the least.

### 4.9. Single Fiber Tensile Test

Tensile properties of fiber predominantly gear on a number of things, like the maturity of plant parts, habitat, fibers chosen for testing, and so on. The presence of cellulose is a crucial factor influencing the mechanical behavior of fiber composites, as it exhibits a diverse range of polymeric actions [71,72]. The tensile strength of the alkalized fiber increased by 192.97 MPa compared to the raw fiber’s value of 92.64 MPa. Additionally, the treated fiber exhibits a high tensile modulus of 3.462 GPa, whereas the raw fiber had 2.164 GPa. The removal of amorphous components resulted in a more organized alignment of microfibrils along the fiber axis, thereby significantly enhancing the strength of the fibers.

Higher MFA (α) might result in poor fiber orientation. The tensile values are on the rise when the MFA is low and vice versa [40]. The elongation at break and strain experienced by the fibers play a crucial role in enhancing the MFA (microfibril angle). Higher MFA introduces higher ductility of fibers, which is also dependent on the orientation of microfibrils. Meanwhile, the MFA (α) of treated fiber (19.67 ± 10.49°) is lower than the raw fiber (21.11 ± 14.08°). The range of MFA values of BP appease with the other fibers and can be introduced for composite reinforcements. A semiempirical relation shown in Equation 9 was formulated by Satyanarayanan et al. It relates to MFA and fiber elongation, and the relation agrees with the BP fibers as well [73].

(9) $$ɛ=2.78+7.28\times 10^{-2}\theta +7.7\times 10^{-3}\theta^{2}$$

where ε is the % elongation, and θ is the MFA with the cellulose content. These tensile values of Butea fibers were compared with various fibers in Table 7.

### 4.10. FTIR Analysis

**Figure 7:**
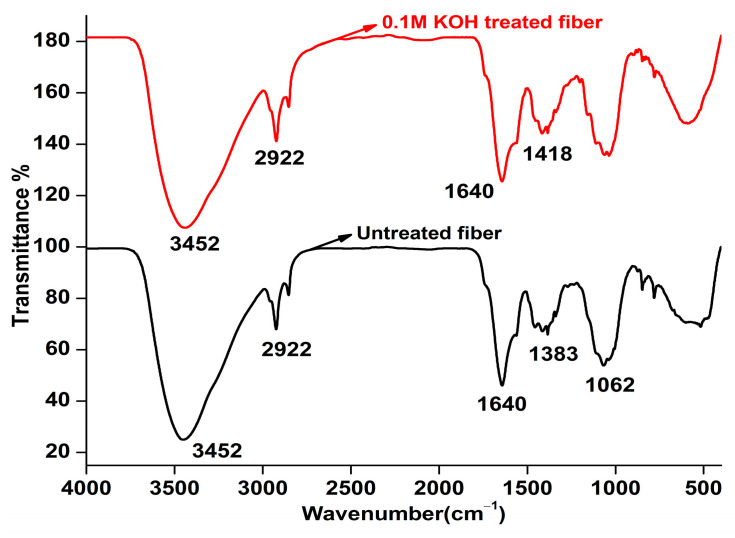
FTIR image of raw and 0.1 M KOH-treated BP.

Spectroscopic investigation on fibers gives a detailed account of the structure and presence of constituents binding with the fiber arrangements like cellulose, hemicellulose, pectin, lignin, and others [78]. The FTIR absorption peak of the raw and treated fibers are provided in Figure 7, and the spectroscopic assignments are listed in Table 8. The presence of a prominent band in the range of 3600–3000 cm^−1^ can be attributed to the stretching of hydrogen-bonded O–H groups in cellulose and/or hemicellulose [79]. A strong peak at 2922 and 2916 cm^−1^ of fibers is the outcome of the C–H stretching vibration of cellulose [80,81]. Due to the free vibration of the carboxyl group, a peak is visible in both fibers at 1640 cm^−1^ [44].

Asymmetric stretching of C–O–C in lignin caused a vibration in the raw fiber at 1383 cm^−1^, whereas the vibration was removed in the treated fibers. Alkaline reagents facilitate the breakdown of lignin into smaller, low-molecular-weight compounds [61,82]. An observable peak split was noticed around 1064 cm^−1^ in the raw BP due to O–H vibrations [83,84]. A glitch noted at 847 cm^−1^ in the raw BP has been unseen in the treated BP. Slight differences in the vibrations of functional groups were observed between the raw and alkalized BP. These variances can be attributed to the removal of specific chemical groups during the alkalization process.

## 5. Conclusions

The aptness of raw and 0.1 M KOH-treated Butea parviflora (BP) fiber to be consumed for green composites was examined, and the following observations were drawn. The density and fineness of the alkalized fiber have risen to (1.34 g/cc) and 346 tex, then the raw fiber, which is (1.23 g/cc) and 312 tex. The chemical composition of fibers clearly witnessed the changes in the levels of cellulose and hemicellulose between the raw and alkalized fibers cellulose hiked to 60.72% while hemicellulose dropped from 40 to 19% in the alkalized BP. The elimination of wax and pectin has a significant impact on the semicrystalline fiber, resulting in enhanced crystallinity. XRD analysis revealed a substantial increase in cellulose content (up to 86.03%) and an enlargement in crystallite size (8.04 nm) after the treatment. FTIR assignments marked slender vibrational changes in the raw and KOH-treated fiber.

The SEM images neatly distinguish the presence and absence of components on the fiber surface, aiding in the analysis of their effective bonding with the matrix phase. Due to their low thermal conductivity (K = 0.020 W/mK), BP fibers are suitable to act as thermal insulators in structural applications. Choosing natural fibers for thermal insulation would significantly reduce carbon footprints compared to synthetic insulators.

Complete analysis of the Tg-dta and DSC studies provided insights into the mass loss of cellulosic and amorphous components at specific temperatures. From the DTG curves, degradation peaks for Butea fibers were observed. The maximum temperature up to which the fibers can stay active was noted to be around 240 °C. The activation energy of the raw fiber (Ea = 73.15 kJ/mol) was higher than that of the treated fiber, indicating that the thermal potentials of the raw fibers are better than the treated BP fibers.

Increments in the crystallinity values and cellulose content directly influence the tensile behavior, showing an abrupt rise in the tensile values of raw BP from 92.64 to 192.97 MPa for the alkalized BP. Only the thermal behavior of raw fibers showed a trifling swiftness than the KOH-treated BP. However, all the properties of treated fibers, except the thermal outcome, surpass those of the untreated fiber.

Summing up the text, the present work highlights the enormity of Butea parviflora fiber through various studies and analyses. The low density, high crystallinity, and thermal stability of BP fibers differentiate its novelty from other green fibers available in the market. It can stand as a suitable contender in the global market of composites in minimizing carbon emissions and safeguarding green territory. The findings provide a positive way to introduce fiber as a reinforcement material in composite making. The impeccable assets of the plant fiber can be further harvested by subjecting them to various treatments along with assessing their properties.

## Figures and Tables

**Table 1:** Comparison made between the physical and chemical attributes of untreated and alkalized BP fibers, alongside other types of fibers.

Fibers	Diameter	Aspect Ratio (L/D)	Linear Density	Density (g/cc)	Reference
Raw BP	0.048 mm	174.59	312 tex	1.238	Present work
Treated BP	0.027 mm	203.91	346 tex	1.340	Present work
Acacia leucophlea	168.5 µm	-	-	1.385	[32]
Coccinia grandis	543–621 µm	-	130.9 tex	1.517	[33]

**Table 2:** Comparison of chemical characteristics of raw and alkalized BP with other fibers.

Fibers	Cellulose (wt%)	Hemicellulose (wt%)	Lignin (wt%)	Moisture (wt%)	Wax (wt%)	Pectin (wt%)
Raw BP	58.5	40.13	18.09	11.63	0.31	6.77
Treated BP	60.72	19.2	20.5	12.4	0.25	3.4
Acacia leucophlea	68.09	13.6	17.73	8.83	-	-
Coccinia grandis	63.22	-	24.42	9.14	0.32	-
Prosopis juliflora bark	61.65	16.14	17.11	9.48	0.61	-

**Table 3:** Comparison of crystallinity index of raw and alkalized BP with other fibers.

Sample	Crystallinity Index (%)	Crystallite Size (nm)	Reference
Untreated BP	83.63	7.50	Present work
Alkali-treated BP	86.03	8.04	Present work
Thespesia populnea	48.17	3.57	[55]
Sida cordifolia stem	56.92	18	[56]

**Table 4:** CHNS analysis of BP fiber.

Sample	N%	C%	H%	S%	Weight (mg)
0.1 M KOH-treated	0.93	41.66	6.60	ND	7.60
Untreated	0.84	39.75	6.30	ND	7.12

ND—not detected.

**Table 5:** Mass loss with temperature from TG.

Fibers	Temperature (°C)	Mass Loss (%)	Residual Char (%)
Raw BP	54–251	17.72	0.4
	251–394	50.78	
	394–540	39.1	
KOH-treated BP	42–209	11.68	2.59
	209–356	45.06	
	356–544	40.67	

**Table 6:** Thermal outcomes of Butea parviflora fibers.

Fibers	Activation Energy (Ea)	Max Degradation Temperature (°C)	Thermal Conductivity (K)
Raw BP	73.15 kJ/mol	365	0.029 W/mk
Alkalized BP	55.95 kJ/mol	324	0.020 W/mk

**Table 7:** Tensile properties of BP and other natural fibers.

Fibers	Tensile Strength (MPa)	Young’s Modulus (GPa)	Elongation at Break (%)	Microfibril Angle (°)	References
Raw BP	92.64	2.164	7.2 ± 3.1	21.11 ± 14.08	Present work
Alkalized BP	192.97	3.462	6.2 ± 1.7	19.67 ± 10.49	Present work
Napier grass	75	6.8	2.8	-	[74]
Cordia dichotoma	36.2	3.6	2.0	-	[75]
Sansevieria ehrenbergii	50–585	1.5–7.67	2.8–21.7	-	[76]
Aerial roots of Banyan	19.37 ± 7.72	1.8 ± 0.40	1.8 ± 0.40	10.88 ± 1.198	[40]
Pennisetum purpureum	73 ± 6	5.68 ± 0.14	1.40 ± 0.23	-	[77]

**Table 8:** Spectroscopic vibrations in BP fibers.

Wavenumber (cm)^−1^		Vibrational Band Assignments
Raw BP	KOH-Treated BP	
3451	3452	O–H stretching with hydrogen bonding in cellulose/hemicellulose
2922	2916	C–H stretching of cellulose
2850	2850	C–H stretching of hemicelluloses
1644	1644	Stretching of C=O in the acetyl group of hemicellulose
-	1418	C–H_2_ symmetric bending in cellulose
1383	-	Asymmetric stretching of C–O–C in lignin
1064	-	C–O and C–C stretching of cellulose
847	-	β-glycosidic linkage in monosaccharides
781	781	CO stretching
517	-	Off-plane OH bending

## Data Availability

Will be provided on request.
